# 4-Chloro-7-hydr­oxy-6-methyl-1,7-naphthyridin-8(7*H*)-one

**DOI:** 10.1107/S1600536809050429

**Published:** 2009-11-28

**Authors:** Kevin D. Bunker, Seiji Nukui, Arnold L. Rheingold, Antonio DiPasquale, Alex Yanovsky

**Affiliations:** a Pfizer Global Research and Development, La Jolla Labs, 10770 Science Center Drive, San Diego, CA 92121, USA; b Department of Chemistry and Biochemistry, University of California, San Diego, 9500 Gilman Drive, La Jolla, CA 92093, USA

## Abstract

The title compound, C_9_H_7_ClN_2_O_2_, was prepared by reaction of methyl 4-chloro-3-(prop-1-yn­yl)picolinate with hydroxy­l­amine in MeOH/KOH solution. The two essentially planar mol­ecules which make up the asymmetric unit have almost identical geometries and and are linked into dimeric aggregates *via* pairs of O—H⋯O hydrogen bonds. These aggregates have almost perfect inversion symmetry; however, quite unusually, the inversion center of the dimer does not coincide with the crystallographic inversion center.

## Related literature

For the synthesis, see: Knight *et al.* (2002 ▶). For the structures of related compounds with a similar bicyclic framework, see: Ikeura *et al.* (1998 ▶); Natsugari *et al.* (1995 ▶). For structural analysis, see: Spek (2009 ▶).
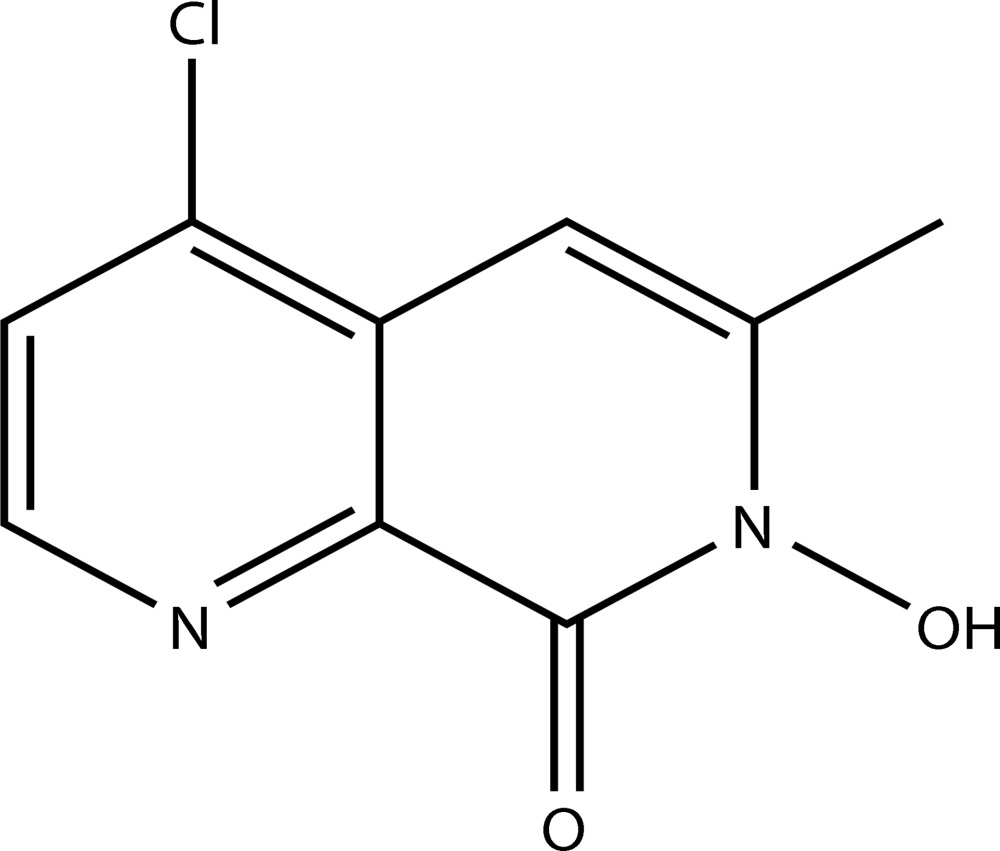


## Experimental

### 

#### Crystal data

C_9_H_7_ClN_2_O_2_
*M* *_r_* = 210.62
Monoclinic, 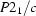

*a* = 9.3983 (4) Å
*b* = 13.8786 (5) Å
*c* = 13.5643 (5) Å
β = 107.663 (3)°
*V* = 1685.86 (11) Å^3^
*Z* = 8
Cu *K*α radiation
μ = 3.80 mm^−1^
*T* = 100 K
0.14 × 0.12 × 0.08 mm

#### Data collection

Bruker APEXII CCD area-detector diffractometer
Absorption correction: multi-scan (*SADABS*; Bruker, 2001 ▶) *T*_min_ = 0.618, *T*_max_ = 0.751
12070 measured reflections
3061 independent reflections
2420 reflections with *I* > 2σ(*I*)
*R*_int_ = 0.032

#### Refinement

*R*[*F* ^2^ > 2σ(*F* ^2^)] = 0.042
*wR*(*F* ^2^) = 0.115
*S* = 1.05
3061 reflections
255 parameters
H-atom parameters constrained
Δρ_max_ = 0.50 e Å^−3^
Δρ_min_ = −0.41 e Å^−3^

### 

Data collection: *APEX2* (Bruker, 2007 ▶); cell refinement: *SAINT* (Bruker, 2007 ▶); data reduction: *SAINT*; program(s) used to solve structure: *SHELXS97* (Sheldrick, 2008 ▶); program(s) used to refine structure: *SHELXL97* (Sheldrick, 2008 ▶); molecular graphics: *SHELXTL* (Sheldrick, 2008 ▶); software used to prepare material for publication: *SHELXTL*.

## Supplementary Material

Crystal structure: contains datablocks global, I. DOI: 10.1107/S1600536809050429/dn2516sup1.cif

Structure factors: contains datablocks I. DOI: 10.1107/S1600536809050429/dn2516Isup2.hkl

Additional supplementary materials:  crystallographic information; 3D view; checkCIF report

## Figures and Tables

**Table 1:** Hydrogen-bond geometry (Å, °)

*D*—H⋯*A*	*D*—H	H⋯*A*	*D*⋯*A*	*D*—H⋯*A*
O11—H11*C*⋯O22	0.84	2.02	2.675 (2)	134
O21—H21*C*⋯O12	0.84	2.09	2.677 (2)	127

**Fig. 1.**
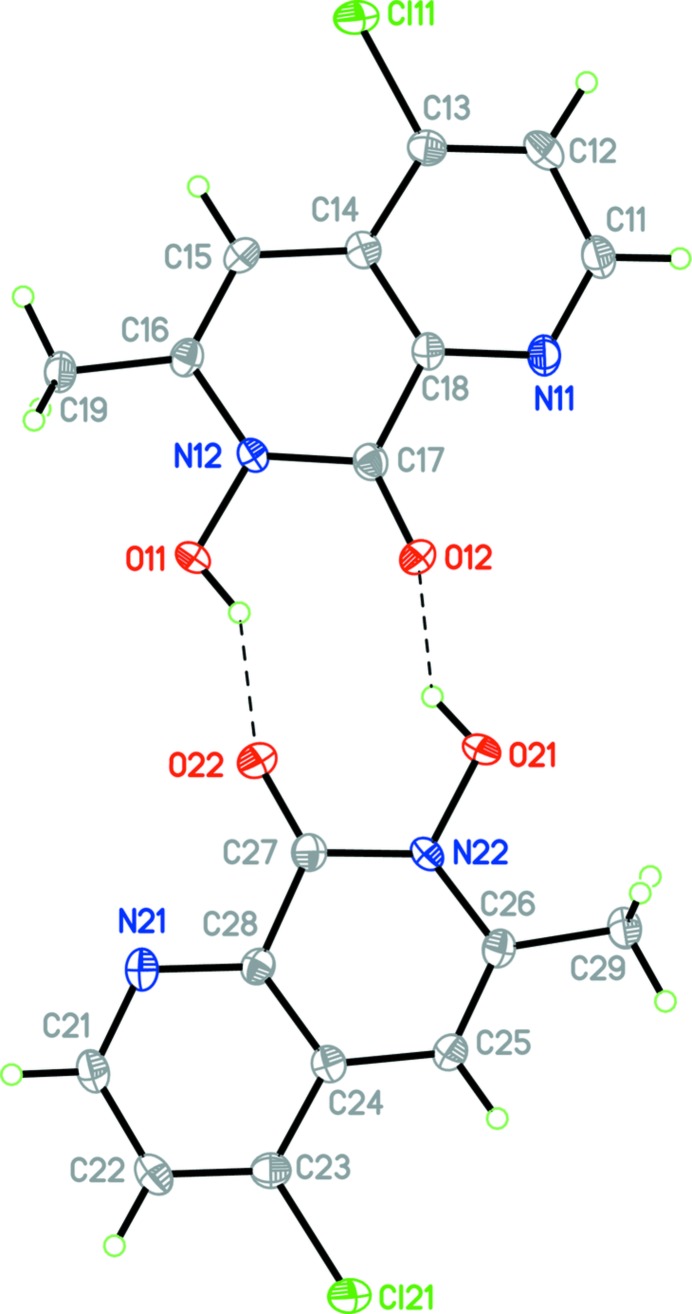
Molecular structure of the title compound, showing 50% probability displacement ellipsoids and atom numbering scheme. H atoms are drawn as circles with arbitrary small radius. H-bonds are shown as dashed lines.

## supplementary crystallographic information

### Comment

The title compound was obtained using the reaction of of methyl 4-chloro-3-(prop-1-ynyl)picolinate with hydroxylamine in MeOH/KOH solution (Knight *et al.*, 2002). The structural formula of the product was confirmed by the present study (Fig. 1).

There are two independent molecules in the structure, which show almost identical geometry. The molecules are essentially planar (with the exception of methyl H atoms) and their parameters are quite similar to those found in related structures with analogous carbon-nitrogen bicyclic framework (Ikeura *et al.*, 1998; Natsugari *et al.*, 1995). To the best of our knowledge, however, this is the first structurally characterized system of this kind with the O-substitution at the N atom next to C=O group.

The molecules in the asymmetric unit of the title compound are linked into dimeric aggregates *via* H-bonds (Table 1). These aggregates have almost ideal inversion symmetry, however, quite unusually, the inversion center of the dimer does not coincide with the crystallographic inversion center.

### Experimental

Warm solutions (50°C) of hydroxylamine hydrochloride (199.0 mg, 2.86 mmol, 6 eq) in methanol (2.0 *M*, 1.43 ml) and potassium hydroxide (241.0 mg, 4.29 mmol, 9 eq) in methanol (4.0 *M*, 1.07 ml) were mixed; the resulting solution was cooled to below 40°C and potassium chloride precipitated out. The precipitate was filtered and the filtrate was added to a vial containing methyl 4-chloro-3-(prop-1-ynyl)picolinate (100.0 mg, 0.4770 mmol); the flask containing the filtrate was rinsed with an additional 1 ml of MeOH and added to the reaction vial. The resulting mixture was then heated to reflux. A precipitate formed within 20 minutes. The reaction was monitored by LCMS; after consumption of starting material (about 75 min), the mixture was removed from heat and cooled to room temperature, diluted with ether and the precipitate was collected. To the precipitate was added minimal amount of acetic acid to quench the mixture. The mixture was then triturated in ethyl acetate and filtered. The filtrate was collected, concentrated and the solid dried to give 26 mg (26%) of the title compound. A small sample was dissolved in methanol:dichloromethane (1:1) and heated at 50°C to dryness to obtain crystals of sufficient quality for X-ray diffraction experiment. LC—MS m/*z* (% relative intensity, ion): 211.0 (100.0%), 213.0 (32.0%), 212.0 (9.9%), 214.0 (3.2%). 1H NMR (400 MHz, DMSO-d6) δ p.p.m. 2.46 (s, 3H) 6.67 (br. s., 1H) 7.88 (br. s., 1H) 8.65 (br. s., 1H) 11.62 (br. s., 1H).

### Refinement

All H atoms were placed in geometrically calculated positions (C—H 0.98 Å and 0.95 Å for methyl and aromatic CH-groups; O—H 0.84 Å) and included in the refinement in riding motion approximation. The *U*_iso_(H) were set to 1.2*U*_eq_ of the carrying atom (1.5*U*_eq_ for methyl and hydroxyl H atoms).

Two independent molecules in the structure of the title compound are related by almost ideal non-crystallographic inversion center, which prompted us to perform additional checks on the presence of higher genuine symmetry by careful inspection of atomic coordinates as well as by using ADDSYM option in *PLATON* (Spek, 2009). Nevertheless, no unaccounted crystallographic symmetry was detected.

### Crystal data

**Table:**

C_9_H_7_ClN_2_O_2_	*F*(000) = 864
*M**_r_* = 210.62	*D*_x_ = 1.660 Mg m^−^^3^
Monoclinic, *P*2_1_/*c*	Cu *K*α radiation, λ = 1.54178 Å
Hall symbol: -P 2ybc	Cell parameters from 4820 reflections
*a* = 9.3983 (4) Å	θ = 4.7–67.9°
*b* = 13.8786 (5) Å	µ = 3.80 mm^−^^1^
*c* = 13.5643 (5) Å	*T* = 100 K
β = 107.663 (3)°	Block, light yellow
*V* = 1685.86 (11) Å^3^	0.14 × 0.12 × 0.08 mm
*Z* = 8	

### Data collection

**Table:**

Bruker APEXII CCD area-detector diffractometer	3061 independent reflections
Radiation source: fine-focus sealed tube	2420 reflections with *I* > 2σ(*I*)
graphite	*R*_int_ = 0.032
phi and ω scans	θ_max_ = 68.1°, θ_min_ = 4.7°
Absorption correction: multi-scan (*SADABS*; Bruker, 2001)	*h* = −11→11
*T*_min_ = 0.618, *T*_max_ = 0.751	*k* = −13→16
12070 measured reflections	*l* = −16→16

### Refinement

**Table:**

Refinement on *F*^2^	Primary atom site location: structure-invariant direct methods
Least-squares matrix: full	Secondary atom site location: difference Fourier map
*R*[*F*^2^ > 2σ(*F*^2^)] = 0.042	Hydrogen site location: inferred from neighbouring sites
*wR*(*F*^2^) = 0.115	H-atom parameters constrained
*S* = 1.05	*w* = 1/[σ^2^(*F*_o_^2^) + (0.0643*P*)^2^ + 0.6521*P*] where *P* = (*F*_o_^2^ + 2*F*_c_^2^)/3
3061 reflections	(Δ/σ)_max_ < 0.001
255 parameters	Δρ_max_ = 0.50 e Å^−^^3^
0 restraints	Δρ_min_ = −0.41 e Å^−^^3^

### Special details

**Table:**

Geometry. All esds (except the esd in the dihedral angle between two l.s. planes) are estimated using the full covariance matrix. The cell esds are taken into account individually in the estimation of esds in distances, angles and torsion angles; correlations between esds in cell parameters are only used when they are defined by crystal symmetry. An approximate (isotropic) treatment of cell esds is used for estimating esds involving l.s. planes.
Refinement. Refinement of *F*^2^ against ALL reflections. The weighted *R*-factor *wR* and goodness of fit *S* are based on *F*^2^, conventional *R*-factors *R* are based on *F*, with *F* set to zero for negative *F*^2^. The threshold expression of *F*^2^ > σ(*F*^2^) is used only for calculating *R*-factors(gt) *etc*. and is not relevant to the choice of reflections for refinement. *R*-factors based on *F*^2^ are statistically about twice as large as those based on *F*, and *R*- factors based on ALL data will be even larger.

### Fractional atomic coordinates and isotropic or equivalent isotropic displacement parameters (Å^2^)

**Table:**

	*x*	*y*	*z*	*U*_iso_*/*U*_eq_	
Cl11	−0.07931 (6)	0.29765 (4)	0.57976 (4)	0.02505 (17)	
O11	0.62329 (17)	0.16036 (11)	0.68417 (13)	0.0229 (4)	
H11C	0.6950	0.1993	0.6968	0.034*	
O12	0.62280 (17)	0.34923 (11)	0.68384 (12)	0.0220 (4)	
N11	0.3605 (2)	0.45541 (13)	0.64550 (14)	0.0199 (4)	
N12	0.4903 (2)	0.21085 (13)	0.66186 (14)	0.0175 (4)	
C11	0.2323 (2)	0.50128 (17)	0.63118 (17)	0.0213 (5)	
H11A	0.2343	0.5697	0.6337	0.026*	
C12	0.0938 (3)	0.45514 (17)	0.61247 (16)	0.0210 (5)	
H12A	0.0051	0.4912	0.6045	0.025*	
C13	0.0903 (2)	0.35705 (17)	0.60603 (16)	0.0186 (5)	
C14	0.2232 (2)	0.30333 (16)	0.62197 (16)	0.0171 (5)	
C15	0.2303 (2)	0.20137 (16)	0.61942 (16)	0.0179 (5)	
H15A	0.1406	0.1649	0.6033	0.021*	
C16	0.3631 (2)	0.15509 (16)	0.63963 (16)	0.0171 (5)	
C17	0.4990 (2)	0.30963 (16)	0.66367 (16)	0.0174 (5)	
C18	0.3550 (2)	0.35808 (16)	0.64246 (16)	0.0166 (5)	
C19	0.3820 (3)	0.04884 (15)	0.63766 (17)	0.0204 (5)	
H19A	0.2837	0.0179	0.6165	0.031*	
H19B	0.4390	0.0263	0.7068	0.031*	
H19C	0.4358	0.0323	0.5884	0.031*	
Cl21	1.58399 (6)	0.29188 (4)	0.92532 (4)	0.02425 (17)	
O21	0.87294 (17)	0.40291 (12)	0.82957 (13)	0.0275 (4)	
H21C	0.8044	0.3616	0.8176	0.041*	
O22	0.88743 (17)	0.21429 (11)	0.81342 (12)	0.0228 (4)	
N21	1.1594 (2)	0.11748 (14)	0.85899 (14)	0.0202 (4)	
N22	1.0090 (2)	0.35700 (13)	0.84478 (14)	0.0187 (4)	
C21	1.2917 (3)	0.07633 (17)	0.87918 (17)	0.0213 (5)	
H21A	1.2960	0.0079	0.8789	0.026*	
C22	1.4260 (3)	0.12739 (17)	0.90091 (17)	0.0221 (5)	
H22A	1.5186	0.0944	0.9153	0.026*	
C23	1.4211 (2)	0.22576 (17)	0.90105 (16)	0.0188 (5)	
C24	1.2824 (2)	0.27445 (16)	0.88048 (16)	0.0163 (5)	
C25	1.2672 (2)	0.37650 (16)	0.88112 (16)	0.0175 (5)	
H25A	1.3531	0.4161	0.8929	0.021*	
C26	1.1319 (3)	0.41747 (16)	0.86517 (17)	0.0180 (5)	
C27	1.0071 (2)	0.25806 (16)	0.83737 (17)	0.0184 (5)	
C28	1.1554 (2)	0.21492 (16)	0.86006 (16)	0.0167 (5)	
C29	1.1054 (3)	0.52295 (16)	0.86981 (18)	0.0225 (5)	
H29A	1.2003	0.5575	0.8825	0.034*	
H29B	1.0629	0.5364	0.9260	0.034*	
H29C	1.0357	0.5443	0.8039	0.034*	

### Atomic displacement parameters (Å^2^)

**Table:**

	*U*^11^	*U*^22^	*U*^33^	*U*^12^	*U*^13^	*U*^23^
Cl11	0.0151 (3)	0.0280 (3)	0.0313 (3)	−0.0015 (2)	0.0058 (2)	0.0012 (2)
O11	0.0132 (8)	0.0163 (8)	0.0371 (9)	0.0040 (6)	0.0046 (7)	0.0022 (7)
O12	0.0165 (8)	0.0176 (8)	0.0311 (9)	−0.0031 (7)	0.0061 (7)	0.0023 (7)
N11	0.0227 (10)	0.0143 (9)	0.0217 (10)	0.0003 (8)	0.0052 (8)	0.0002 (8)
N12	0.0155 (10)	0.0133 (9)	0.0238 (10)	0.0022 (7)	0.0059 (8)	0.0011 (8)
C11	0.0249 (12)	0.0154 (11)	0.0222 (11)	0.0031 (10)	0.0050 (9)	−0.0002 (9)
C12	0.0209 (12)	0.0219 (12)	0.0201 (11)	0.0072 (10)	0.0059 (9)	−0.0002 (9)
C13	0.0173 (11)	0.0207 (12)	0.0172 (11)	0.0007 (9)	0.0041 (9)	0.0005 (9)
C14	0.0190 (12)	0.0173 (11)	0.0163 (11)	0.0008 (9)	0.0070 (9)	0.0007 (9)
C15	0.0171 (11)	0.0174 (11)	0.0189 (11)	−0.0040 (9)	0.0050 (9)	−0.0010 (9)
C16	0.0199 (11)	0.0154 (11)	0.0156 (10)	−0.0022 (9)	0.0048 (9)	0.0007 (9)
C17	0.0188 (11)	0.0163 (11)	0.0177 (11)	0.0012 (9)	0.0062 (9)	0.0019 (9)
C18	0.0189 (12)	0.0139 (11)	0.0167 (11)	0.0002 (9)	0.0051 (9)	0.0001 (9)
C19	0.0230 (11)	0.0133 (11)	0.0223 (11)	−0.0006 (9)	0.0033 (9)	−0.0001 (9)
Cl21	0.0159 (3)	0.0256 (3)	0.0303 (3)	−0.0007 (2)	0.0057 (2)	0.0016 (2)
O21	0.0126 (8)	0.0200 (8)	0.0473 (11)	0.0039 (7)	0.0053 (7)	−0.0038 (8)
O22	0.0171 (8)	0.0211 (8)	0.0297 (9)	−0.0029 (7)	0.0063 (7)	0.0010 (7)
N21	0.0248 (10)	0.0143 (9)	0.0217 (10)	0.0000 (8)	0.0074 (8)	−0.0010 (8)
N22	0.0150 (9)	0.0149 (10)	0.0255 (10)	0.0037 (8)	0.0051 (8)	−0.0004 (8)
C21	0.0255 (12)	0.0147 (11)	0.0234 (12)	0.0033 (10)	0.0069 (10)	−0.0004 (9)
C22	0.0231 (12)	0.0199 (12)	0.0244 (12)	0.0065 (10)	0.0089 (10)	0.0018 (10)
C23	0.0172 (11)	0.0221 (12)	0.0167 (11)	0.0000 (9)	0.0045 (9)	0.0000 (9)
C24	0.0188 (12)	0.0163 (11)	0.0138 (11)	0.0012 (9)	0.0050 (9)	0.0009 (9)
C25	0.0184 (11)	0.0164 (11)	0.0174 (11)	−0.0018 (9)	0.0047 (9)	−0.0005 (9)
C26	0.0204 (11)	0.0149 (11)	0.0191 (11)	−0.0014 (9)	0.0065 (9)	0.0012 (9)
C27	0.0191 (12)	0.0170 (11)	0.0190 (11)	0.0000 (9)	0.0057 (9)	0.0017 (9)
C28	0.0182 (12)	0.0161 (11)	0.0166 (11)	−0.0014 (9)	0.0061 (9)	−0.0010 (9)
C29	0.0236 (12)	0.0155 (12)	0.0268 (12)	0.0013 (10)	0.0053 (10)	0.0001 (10)

### Geometric parameters (Å, °)

**Table:**

Cl11—C13	1.733 (2)	Cl21—C23	1.729 (2)
O11—N12	1.384 (2)	O21—N22	1.387 (2)
O11—H11C	0.8400	O21—H21C	0.8405
O12—C17	1.240 (3)	O22—C27	1.232 (3)
N11—C11	1.323 (3)	N21—C21	1.320 (3)
N11—C18	1.352 (3)	N21—C28	1.353 (3)
N12—C17	1.373 (3)	N22—C27	1.377 (3)
N12—C16	1.378 (3)	N22—C26	1.386 (3)
C11—C12	1.403 (3)	C21—C22	1.399 (3)
C11—H11A	0.9500	C21—H21A	0.9500
C12—C13	1.364 (3)	C22—C23	1.366 (3)
C12—H12A	0.9500	C22—H22A	0.9500
C13—C14	1.414 (3)	C23—C24	1.419 (3)
C14—C18	1.407 (3)	C24—C28	1.408 (3)
C14—C15	1.418 (3)	C24—C25	1.424 (3)
C15—C16	1.356 (3)	C25—C26	1.349 (3)
C15—H15A	0.9500	C25—H25A	0.9500
C16—C19	1.486 (3)	C26—C29	1.489 (3)
C17—C18	1.459 (3)	C27—C28	1.462 (3)
C19—H19A	0.9800	C29—H29A	0.9800
C19—H19B	0.9800	C29—H29B	0.9800
C19—H19C	0.9800	C29—H29C	0.9800
			
N12—O11—H11C	109.5	N22—O21—H21C	109.5
C11—N11—C18	116.9 (2)	C21—N21—C28	117.2 (2)
C17—N12—C16	127.42 (19)	C27—N22—C26	127.62 (19)
C17—N12—O11	117.17 (18)	C27—N22—O21	117.13 (18)
C16—N12—O11	115.41 (17)	C26—N22—O21	115.25 (17)
N11—C11—C12	124.1 (2)	N21—C21—C22	123.9 (2)
N11—C11—H11A	118.0	N21—C21—H21A	118.0
C12—C11—H11A	118.0	C22—C21—H21A	118.0
C13—C12—C11	118.1 (2)	C23—C22—C21	118.5 (2)
C13—C12—H12A	121.0	C23—C22—H22A	120.7
C11—C12—H12A	121.0	C21—C22—H22A	120.7
C12—C13—C14	120.9 (2)	C22—C23—C24	120.4 (2)
C12—C13—Cl11	119.42 (18)	C22—C23—Cl21	120.15 (18)
C14—C13—Cl11	119.71 (18)	C24—C23—Cl21	119.49 (18)
C18—C14—C13	115.4 (2)	C28—C24—C23	115.6 (2)
C18—C14—C15	119.9 (2)	C28—C24—C25	120.3 (2)
C13—C14—C15	124.6 (2)	C23—C24—C25	124.1 (2)
C16—C15—C14	121.0 (2)	C26—C25—C24	120.7 (2)
C16—C15—H15A	119.5	C26—C25—H25A	119.7
C14—C15—H15A	119.5	C24—C25—H25A	119.7
C15—C16—N12	117.5 (2)	C25—C26—N22	117.7 (2)
C15—C16—C19	125.0 (2)	C25—C26—C29	124.7 (2)
N12—C16—C19	117.43 (19)	N22—C26—C29	117.6 (2)
O12—C17—N12	119.6 (2)	O22—C27—N22	120.1 (2)
O12—C17—C18	126.2 (2)	O22—C27—C28	126.1 (2)
N12—C17—C18	114.20 (19)	N22—C27—C28	113.74 (19)
N11—C18—C14	124.6 (2)	N21—C28—C24	124.4 (2)
N11—C18—C17	115.49 (19)	N21—C28—C27	115.7 (2)
C14—C18—C17	119.9 (2)	C24—C28—C27	119.9 (2)
C16—C19—H19A	109.5	C26—C29—H29A	109.5
C16—C19—H19B	109.5	C26—C29—H29B	109.5
H19A—C19—H19B	109.5	H29A—C29—H29B	109.5
C16—C19—H19C	109.5	C26—C29—H29C	109.5
H19A—C19—H19C	109.5	H29A—C29—H29C	109.5
H19B—C19—H19C	109.5	H29B—C29—H29C	109.5

### Hydrogen-bond geometry (Å, °)

**Table:**

*D*—H···*A*	*D*—H	H···*A*	*D*···*A*	*D*—H···*A*
O11—H11C···O22	0.84	2.02	2.675 (2)	134
O21—H21C···O12	0.84	2.09	2.677 (2)	127
